# Associations between dimensions of empowerment and nutritional status among married adolescent girls in East Africa: a structural equation modelling study

**DOI:** 10.1186/s12889-022-14949-1

**Published:** 2023-02-02

**Authors:** Alison Y. Riddle, Wenshan Li, Zulfiqar A. Bhutta, Carol Vlassoff, Monica Taljaard, Elizabeth Kristjansson, Vivian Welch, George A. Wells

**Affiliations:** 1 School of Epidemiology and Public Health, University of Ottawa, 600 Peter Morand Crescent, Room 101, Ottawa, ON K1G 5Z3 Canada; 2 Bruyère Research Institute, 85 Primrose Avenue, Ottawa, ON K1R 7G5 Canada; 3 Centre for Global Child Health, Hospital for Sick Children (SickKids), 525 University Avenue, Suite 702, Toronto, ON M5G 2L3 Canada; 4 Institute for Global Health and Development, Aga Khan University, Stadium Road, P.O. Box 3500, Karachi, 74800 Pakistan; 5 Clinical Epidemiology Program, Ottawa Hospital Research Institute, 501 Smyth Road Box 511, Ottawa, ON K1H 8L6 Canada; 6 School of Psychology, Social Sciences Building, University of Ottawa, 120 University Private, Ottawa, ON K1N 6N5 Canada; 7 University of Ottawa Heart Institute, 40 Ruskin Street, Ottawa, ON K1Y 4W7 Canada

**Keywords:** Adolescent Girls, Adolescent Nutrition, Adolescent Empowerment, East Africa, Structural Equation Models

## Abstract

**Background:**

Empowering adolescent girls is an important component of combating malnutrition in this age group. Because empowerment is multidimensional and context specific, it can be difficult for policymakers and practitioners to target the dimensions of empowerment associated with adolescent girls’ nutrition in a particular setting. This study sought to identify the empowerment dimensions significantly associated with married adolescent girls' nutritional status in East Africa; a region where malnutrition and gender inequality stubbornly persist.

**Methods:**

We used cross-sectional Demographic and Health Survey (DHS) data from Ethiopia (2016), Kenya (2014), Tanzania (2015–16) and Uganda (2016) to construct and test theoretically informed structural equation models of the relationship between six dimensions of empowerment and BMI-for-age and haemoglobin levels for married adolescent girls aged 15–19 years.

**Results:**

Our models were found to be a good fit for the data. Married adolescent girls’ access to information, measured by their education level and mass media use, was directly and positively associated with their BMI-for-age (*p* < 0.05). Asset ownership, measured by owning a house or land alone or jointly, was directly and positively associated with haemoglobin (*p* < 0.05) and reduced odds of being moderately to severely anemic. Rejecting justifications for intimate partner violence, a measure of respondents’ intrinsic agency, was directly and positively associated with the odds of being overweight or obese. Adolescent girls’ level of empowerment across all dimensions had a direct relationship with their country of residence and household wealth.

**Conclusions:**

Our findings suggest that investment in girls’ access to information through education and mass/social media and their economic empowerment may be important contributors to their overall empowerment and nutritional status. However, caution is needed as greater autonomy may contribute to increased consumption of unhealthy foods that can contribute to overweight and obesity. Strategies to empower married adolescent girls should be tailored to their specific circumstances. There is an urgent need for better data on adolescent empowerment and health, including increased research into age-, sex- and gender-appropriate empowerment measures and longitudinal data to assess causality. The use of statistical models should be complemented by robust qualitative research to further results interpretation.

**Supplementary Information:**

The online version contains supplementary material available at 10.1186/s12889-022-14949-1.

## Background

Late adolescence, from the ages of 15 to 19 years, is a time of important physiological growth and neurodevelopment for girls that can be undermined by poor nutrition. Bone acquisition continues into late adolescence, with final bone mineral content plateauing in girls at around 18 years [1]. Undernutrition in late adolescence can have negative consequences on brain development while chronic malnutrition increases the risk of adiposity and non-communicable diseases later in life [1]. Adolescent pregnancy has several negative consequences for adolescent girls’ health and contributes to an intergenerational cycle of malnutrition [2].

East Africa is a region where progress on addressing malnutrition among adolescent girls is slow. In the last decade, thinness (BMI-for-age (ZBFA) < -2 standard deviations (SD)) among girls aged 5–19 years in East Africa declined only 0.5 to 4.6%, while the prevalence of overweight and obesity (ZBFA > 1 SD) increased from 5.5 to 17.3% over the same period [3]. Approximately 32% of all women and girls aged 15–49 years in the region are anemic [3].

Sex- and gender-related disparities in nutrition are a consequence of physiological and sociocultural factors. Iron-deficiency anemia affects both sexes, but adolescent girls carry the greatest burden due to the additional iron requirements that come with the onset of menarche. Boys acquire more lean body mass during adolescence while girls accrue more fat body mass, contributing to higher rates of thinness in boys and overweight and obesity in girls [1]. Early and child marriage contributes to adolescent pregnancy which accounts for over 10% of global total births annually [4]. The competition for nutrients between the still growing mother and the fetus restricts maternal growth and increases the risk of preterm delivery, low birthweight, and small-for-gestational age among neonates [5]. Son preference in families results in many girls eating last, least, and of lesser quality compared to boys and men in the household [6]. It also de-prioritizes their timely access to healthcare needed to prevent disease and unplanned pregnancy.

The drivers of malnutrition among adolescents are multifactorial. Individual behaviours such as food choice, dietary intake and physical activity are influenced by the food environment which is shaped by household and community dietary and activity patterns, economic development, urbanization and food and agriculture systems [7]. Gender inequality and restrictive gender norms play an important role in shaping these socio-ecological systems and disproportionately contribute to women and girls’ poor health [7, 8]. Rigid gender norms are absorbed as early as age 10, coinciding with a shift in boys and girls’ roles in the family and community on gendered lines [9]. For girls, this often means a decline in their agency and autonomy relative to boys, yet agency, or their ability to define and achieve goals, is critical to adolescent well-being [10]. The empowerment of adolescent girls is an important component of combating malnutrition in this age group [11–13]. Empowering married adolescent girls may require special attention compared to adult women. This group can face additional challenges to their agency and autonomy due to their relatively young age and lower status in the household. Younger age at marriage is associated with decreased bargaining power in the household [14]. Large age gaps between child brides and their husbands further disempower adolescent girls in the household hierarchy [15]. Married adolescent girls are more likely to become pregnant and prematurely leave school, stunting their learning and limiting their economic prospects [16]. Child marriage itself is a disempowering action and a human rights violation as girls often have little say in the decision of when and who to marry [17].

Empowerment is a complex construct that is multidimensional and context specific. As such, it can be difficult for policy makers and practitioners to identify and target the dimensions of empowerment that may positively influence adolescent girls’ nutrition in a particular setting. A recent systematic review of women’s empowerment measures in developing countries found the most common dimensions measured were household decision making, financial and economic decision making, freedom of movement, self-esteem, and sexual and reproductive decision making [18]. Further, there is evidence that different dimensions of women’s empowerment influence maternal and child nutrition differently [19–22]. We hypothesized that a similar phenomenon exists for married girls in late adolescence, but there may be differences as to which empowerment dimensions are significant given this population’s particular nutritional and empowerment-related challenges. Compared to adult women, the relationship between married adolescent girls’ empowerment and their nutritional status is understudied. As such, the objective of this study was to model the relationship between dimensions of married adolescent girls’ empowerment and their nutritional status using cross-sectional Demographic and Health Survey data from four East African countries (Ethiopia, Kenya, Tanzania, and Uganda).

## Methods

### Conceptualizing empowerment

Empowerment refers to “the process by which those who have been denied the ability to make strategic life choices acquire such an ability” [23]. It involves a progression from a position of lacking choice, to once in which choice is exercised. Most importantly, it reflects *agency*, or the “ability to define one’s goals and act upon them,” in important life areas [23]. Decisions regarding strategic life choices have long term consequences for a person’s life course and well-being, such as those regarding their education, reproductive health, and economic prospects. Agency over strategic life choices contributes to the realization and protection of individuals’ human rights.

Empowerment is a complex and multidimensional construct. People can be empowered or disempowered in different area of their lives, which we term, *dimensions*. For example, a married adolescent girl may have the ability to choose what food is cooked daily, but she may not make decisions regarding contraceptive use. The ability to make strategic life choices across different dimensions reflects a person’s *capabilities*. Capabilities are ways of ‘doing or being,’ or what people are actually able to be or do in different life areas that are important to them [24, 25].

A person’s ability to access and control resources is crucial to the empowerment process. Kabeer identifies resources as necessary preconditions to the exercise of agency [23]. Resources are defined broadly to encompass not only economic or financial resources, such as earning an income or owning assets, but also human and social capital, time, and information. The more disempowered an individual, the greater likelihood that they have limited resources at their disposal. An individual’s access to resources is shaped by their surrounding political, economic, legal, and socio-cultural environment. Alsop and Heinsohn (2005) define this concept as a person’s *opportunity structure*, or the formal and informal institutions including laws, regulatory frameworks, and norms that define people’s behaviours. They state that “[t]he presence and operation of the formal and informal laws, regulations, norms, and customs determine whether individuals and groups have access to assets, and whether these people can use the assets to achieve their desired outcomes” [26].

**Fig. 1:**
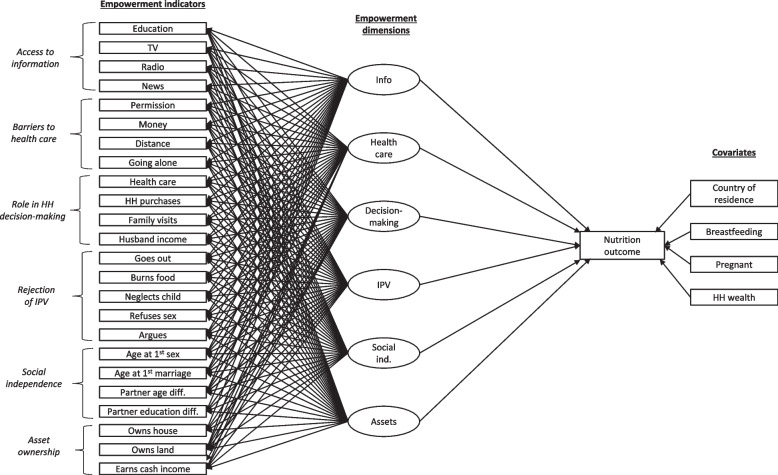
Exploratory structural equation model of the relationship between dimensions of empowerment and nutrition status, controlling for important covariates. Notes: Rectangles indicate observed (directly measured) variables. Ovals represent latent constructs or factors (empowerment dimensions). Unidirectional arrows represent direct effects of exposure on outcome. The lefthand side of the model is an exploratory factor analysis of empowerment indicators to define the underlying factor structure (empowerment dimensions). The righthand side of the model is a regression analysis of a nutritional outcome on empowerment dimensions. Both sides of the model are estimated simultaneously. Not shown: Error terms for estimated variables, covariance of factors

Our conceptual model (Fig. 1) brings together these components of empowerment to describe a process whereby agency, resources, and opportunity structure work together to create individual capabilities. These capabilities across different dimensions of empowerment are proposed to contribute to the achievement of an individual’s goals. Specifically, our model theorizes that adolescent girls’ capabilities across six empowerment dimensions have a direct influence on their nutritional status (Fig. 1). The dimensions are: (1) the ability to access information, (2) the ability to access healthcare, (3) the ability to make household decisions, (4) the ability to reject intimate partner violence, (4) the ability to own assets, and (6) the ability to exert social independence. The empowerment dimensions are measured (imperfectly) as latent constructs using observed indicators from the DHS. We reviewed existing studies (see Additional File 1) that modelled the nutrition-empowerment relationship using DHS data to identify indicators and empowerment dimensions to include in our model. We sought to identify indicators that measured not only the ability to make decisions (a direct measure of agency), but also measures of adolescent girls’ access to resources and the existence of a supportive opportunity structure that would promote adolescent girls’ agency and empowerment. Indicators selected for inclusion had to be (1) available across datasets with the percentage of missing data close to zero, (2) previously used as a measure of empowerment, (3) have sufficient variation to have adequate explanatory power, (4) be theoretically justified, and (5) contain no indicator correlations greater than 0.95 [27, 28]. Details on indicator coding and scales are available in Additional File 1.

### Dimension 1: access to information

Access to information is necessary for adolescent girls to make informed decisions about their health and nutrition. We identified four relevant DHS indicators that we hypothesized would measure this construct. The first indicator was adolescent girls’ level of completed education (measured in years). Education level is a strong predictor of health and nutrition among women and girls [29]. The remaining indicators measured respondents’ use of mass media. The DHS asks respondents about the frequency in which they (1) watched television, (2) read the newspaper or magazine, and (3) listened to the radio. The use of mass media in behaviour change interventions has been shown to positively influence health-related knowledge and attitudes among adolescents and has the potential to positively shape gender norms alongside social media and technology [30, 31].

### Dimension 2: access to healthcare

Adolescent girls can face various barriers to healthcare, many of which are linked to gender norms and inequality. Limited access to healthcare contributes to their increased risk of illness, disease, and unplanned pregnancy—all important contributors to malnutrition in this population. The DHS includes four variables to assess respondents’ ability to access healthcare by asking them to indicate whether the following barriers to healthcare are “a big problem/not a big problem”: (1) getting permission to go, (2) not wanting to go alone, (3) getting money for treatment, and (4) distance to the health facility.

### Dimension 3: role in household decision-making

Decision-making power is frequently used as a measure of agency. The DHS includes four indicators to assess married respondents’ level of participation in household decision-making (either not at all, alone, or jointly with their partner/other household member) in the following situations: (1) when the respondent wants to seek healthcare for themselves, (2) when they want to visit family or relatives, (3) when large household purchases are made, and (4) when decisions about their husband's income are made.

### Dimension 4: rejecting IPV

Adolescent girls’ views on intimate partner violence reflect the degree to which they have internalized harmful gender norms. Other studies have used these indicators to measure women’s intrinsic agency [32–34]. The DHS asks respondents whether they agree (yes/no) that a husband is justified in beating his wife in the following scenarios: (1) when she neglects the children, (2) when she burns food, (3) when she refuses sex with her husband, (4) when she goes out without telling her husband, and (5) when she argues with her husband.

### Dimension 5: asset ownership

Access to assets was hypothesized to capture respondents’ financial autonomy and their ability to access and control financial resources. Three indicators were included in this dimension: (1) whether the respondent owns a house either alone or jointly, (2) whether the respondent owns land either alone or jointly and (2) whether the respondent earns a cash income.

### Dimension 6: social independence

The final dimension examined respondents’ ability to counter negative social norms that disempower women and girls, such as child/early/forced marriage and adolescent pregnancy. It included (1) respondent’s age at first cohabitation (a higher age was interpreted as greater empowerment) [35], (2) respondent’s age at first sex (early sexual activity is often due to a lack of choice and further disempowers girls’ when adolescent pregnancy leads to school dropouts) [36], (3) respondent’s age difference with her partner (smaller gap = greater empowerment), and (4) her education level difference with her partner (smaller gap = greater empowerment).

### Data and study sample

The study population was adolescent girls in late adolescence (15–19 years) who (1) were surveyed as part of the most recent DHS VII surveys conducted in Ethiopia, Kenya, Tanzania, and Uganda, (2) were selected for anthropometric and anemia testing, and (3) reported being currently partnered (married) at the time of the survey.

The DHS is a nationally representative cross-sectional survey of women of reproductive age (15–49 years) and men aged 15–49 years, 15–54 years, or 15–59 years. The survey sample is based on a stratified two-stage cluster design that is typically representative at the national level, at the residence level (urban/rural), and the regional level (e.g., states). The DHS uses standardized questionnaires and has large sample sizes (usually between 5,000 and 30,000 households). The surveys are conducted in low- and middle-income countries with support from the U.S. government approximately every five years to allow for comparisons across countries and time.

The DHS is one of the only data sources with cross-country comparable quantitative data on older adolescent girls’ (15–19 years) nutrition and empowerment. While the module is not specifically designed for adolescent girls, it can still provide insights into their empowerment and nutrition. The DHS women’s status module assesses female respondents’ ability to participate in household decision-making (collected for married respondents only), their attitudes toward gender roles, their employment, and their ownership of a house or land.

### Outcome measures

We assessed the direct association of dimensions of empowerment on adolescent girls’ BMI-for-age (z-score) and haemoglobin levels (Hb) (g/dl). A separate model was tested for each nutrition outcome. Z-scores were calculated using the 2007 WHO growth standards for age and sex [37]. Haemoglobin values were adjusted for altitude and smoking status [38]. Furthermore, we dichotomized respondents’ BMI-for-age and haemoglobin outcomes to construct and test models for the following: (1) thinness (ZBFA < -2 SD), (2) anemia (Hb < 12.0 g/dl for non-pregnant respondents and Hb < 11.0 g/dl for pregnant respondents), (3) moderate to severe anemia (Hb < 11.0 g/dl for non-pregnant respondents and Hb < 10.0 g/dl for pregnant respondents), and (4) overweight/obesity (ZBFA > 1 SD).

### Covariates

Independent variables for adolescent girls’ country of residence, household wealth, breastfeeding status and pregnancy status were included in the model to control for their effects on nutritional status [39–42]. We selected covariates based on a review of similar studies, the content and methodological expertise of the author team, and variable availability in the DHS. The BMI-for-age model controlled for the current breastfeeding status of respondents (yes, no), household wealth (measured as a continuous composite measure of a household’s cumulative wealth as part of the DHS), and country of residence. Pregnant respondents were omitted from the BMI-for-age model. We included country of residence as a proxy measure of engrained rules, norms, and practices that shape choice for adolescent girls [23]. Effect coding was used to create dummy variables for country of residence whereby ones, zeros, and negative ones were used to assign group (country) membership [43]. Indicator values represented the deviation of the country value from the unweighted grand mean (UGM) of the outcome variable. The Hb model included the same covariates and controlled for current pregnancy status (yes, no).

### Model development and testing

We employed exploratory structural equation modelling (ESEM) with covariates to construct and test our model. Structural equation modelling (SEM) is a group of statistical procedures used for causal inference that tests hypotheses regarding the linear relations among observed variables and latent constructs (or factors) [27]. SEM uses a combination of factor analysis and multiple regression analysis and is appropriate for research questions that involve complex, multifaceted constructs measured with error, and to specify systems of relationships with direct and indirect effects [44]. Because empowerment is multi-dimensional and cannot be directly measured, SEM offers a viable alternative to multiple regression because it can appropriately accommodate multiple indicators measuring a latent construct with measurement error. The basic datum analysed in SEM is covariance, which represents the strength of the linear association between two variables. The observed variables can be continuous, censored, binary, ordered categorical (ordinal), and combinations thereof [45]. The goals of SEM are to understand the patterns of covariance among a set of observed variables and to explain as much of their variance as possible with the model. Exploratory structural equation modelling (ESEM) is a less restrictive SEM approach that allows observed variables to (minimally) cross-load onto more than one factor instead of restricting factor loadings to zero on all other factors it is not designed to measure. Such a restriction has been shown to be unrealistic in real world settings and contribute to the inflation of factor correlations and biased estimates [46].

We built the model in a stepwise manner, beginning with an exploratory factor analysis (EFA) of the 24 empowerment indicators (see left-hand side of Fig. 1) using Mplus Version 8 [45]. The Mean- and Variance-Adjusted Weighted Least Squares (WLSMV) estimator was used, and an oblique (geomin) rotation was applied. A series of 1 to 10 factor models were run. Complex survey design was incorporated using TYPE = COMPLEX in the Analysis command along with variables for stratification, weighting, and clustering, as per the DHS survey design for each country. Empowerment indicators were retained if their primary factor loading was above 0.40, below 0.30 for alternative factors, and theoretically justified [47]. Model fit was assessed using recommended model fit indices and corresponding cut-offs [44]. The number of extracted factors was determined by model fit values, scree plot inspection and Eigenvalue > 1, factor interpretability, and parsimony. Factor quality was assessed by examining the number of indicators that loaded on each factor and the correlation between the estimated factor score and the factor [44].

Once a stable EFA structure with good fit was obtained, a nutrition outcome was added to the model to assess the direct effect of each factor (empowerment dimension) on the outcome of interest using regression analysis (right-hand side of Fig. 1). Covariates were added in the following step and model stability and fit were re-assessed. As a final step, modification indices were checked to identify missing paths that were theoretically justified, and improved model fit [44]. Indicators that contributed to a not positive definite residual covariance matrix were investigated and, where appropriate, removed from the model. The model was then re-run. Separate models were run for BMI-for-age, Hb, thinness (ZBFA < -2 SD), overweight and obesity (ZBFA > 1 SD), anemia (Hb < 12.0 g/dl for non-pregnant respondents and Hb < 11.0 g/dl for pregnant respondents), and moderate to severe anemia (Hb < 11.0 g/dl for non-pregnant respondents and Hb < 10.0 g/dl for pregnant respondents). Statistical significance was determined at the α < 0.05 level. The unit of analysis was the individual respondent.

## Results

### Study population characteristics

The total sample size was 1,927 married adolescent girls (Table 1). The mean age of respondents was 17.9 years (SD = 1.1). Most respondents were from the two poorest socio-economic quintiles (57.6%) and lived in rural settings (80.2%). Education levels varied by country, with Ethiopia having the lowest mean level of completed education (3.8 years, SD = 3.5), and Kenya the highest (6.5 years, SD = 3.6). Almost 22% of respondents were pregnant and 41.2% were breastfeeding at the time of the surveys. The mean BMI of non-pregnant respondents was 20.8 kg/m^2^. The prevalence of thinness and overweight (including obesity) among non-pregnant respondents was 3.5 and 7.0%, respectively. Almost 44% of respondents with available data were anemic.

**Table 1:** Demographic characteristics and nutritional status of married adolescent girls (15–19 years) from Demographic and Health Surveys conducted in Ethiopia (2016), Kenya (2014), Tanzania (2015–16), and Uganda (2016)

Variable	Regional (*n* = 1,927)	Ethiopia (*n* = 664)	Kenya (*n* = 352)	Tanzania (*n* = 619)	Uganda (*n* = 292)
Mean age (SD)	17.9 (1.1)	17.7 (1.1)	18.0 (1.1)	17.9 (1.2)	18.1 (1.0)
Mean level of completed education (SD)	5.1 (3.4)	3.8 (3.5)	6.5 (3.6)	5.4 (3.0)	5.9 (2.5)
Living in an urban setting, % (n)	19.8 (381)	16.7 (111)	33.8 (119)	17.6 (109)	14.4 (42)
Household wealth below the middle quintile, % (n)	57.6 (1,109)	56.3 (374)	60.8 (214)	55.7 (345)	60.3 (176)
Currently pregnant, % (n)	21.6 (416)	16.1 (107)	22.7 (80)	25.5 (158)	24.3 (71)
Currently breastfeeding, % (n)	41.7 (804)	33.1 (220)	50.3 (175)	43.6 (270)	47.6 (139)
Anemic (Hb < 12.0 g/dl, < 11.0 g/dl (preg)), % (n) ^a^	43.8 (664)	32.3 (198)	NA	57.1 (350)	40.1 (116)
Mean BMI (SD) ^b^	20.8 (3.1)	19.7 (3.0)	21.0 (3.1)	21.6 (3.3)	21.4 (2.4)
Thin (ZBFA < -2SD), % (n)	3.5 (52)	7.8 (41)	2.6 (7)	0.9 (4)	0.0 (0)
Overweight or obese (ZBFA > 1SD), % (n)	7.0 (103)	3.0 (16)	9.9 (27)	9.0 (41)	8.7 (19)

*BMI* Body Mass Index, *NA* Not Available. Kenya DHS (2014) did not conduct anemia testing, *SD* Standard Deviation, *ZBFA* BMI-for-age z-score

^a^ Total sample size for anemia testing = 1,515

^b^ Total sample size for anthropometric measures = 1,476

Fifty-five percent of respondents reported listening to the radio while only 28.5% watched TV and 18.9% read the newspaper (Table 2). The highest reported barriers to healthcare were money (41.6%) and distance (44.4%). Respondent’s average age at first cohabitation and first sex were 16.1 years and 15.5 years, respectively. Just over 28% earned some cash income. Over 39% owned a house and 32.9% owned land, either alone or jointly. Respondents had the greatest role in household decision-making regarding their own healthcare (67.6%) and the lowest influence on decisions regarding large household purchases (52.3%). Most respondents rejected the view that husbands were justified in beating their wives if they burn food (72.9%), but a majority (51.6%) agreed that violence is justified if a wife neglects the children.

**Table 2:** Empowerment indicator means, proportions, and factor loadings^a^

Indicator name	Mean value or proportion ‘empowered’ *n* = 1,927	F1	F2	F3	F4	F5	F6
Dimension 1: Access to information							
Completed level of education, mean (SD)	5.1 (3.4)	**0.570**	0.050	0.032	0.084	-0.072	0.150
Watches TV, % (n)	28.5 (549)	**0.635**	0.117	0.039	0.004	-0.091	-0.013
Reads newspaper or magazine, % (n)	18.9 (364)	**0.763**	-0.181	0.016	-0.011	0.003	0.048
Listens to the radio, % (n)	55.0 (1060)	**0.771**	0.025	-0.155	0.004	0.060	-0.035
Dimension 2: Barriers to healthcare							
Getting permission, % (n)	81.4 (1569)	0.002	**0.876**	-0.070	0.017	0.040	0.014
Money, % (n)	54.8 (1056)	0.074	**0.737**	0.021	-0.016	-0.010	-0.024
Distance, % (n)	55.6 (1072)	0.020	**0.878**	0.046	-0.042	-0.037	-0.016
Going alone, % (n)	69.4 (1337)	-0.027	**0.840**	-0.007	0.046	0.024	0.035
Dimension 3: Social independence							
Age at 1^st^ marriage, mean (SD)	16.0 (1.6)	0.053	0.004	-0.038	0.008	0.011	**0.895**
Age at 1^st^ sex, mean (SD)	15.5 (1.7)	0.051	-0.012	0.057	-0.023	0.001	**0.693**
Partner age difference, mean (SD)	-7.4 (4.6)	E	E	E	E	E	E
Partner education difference, mean (SD)	-1.1 (3.7)	E	E	E	E	E	E
Dimension 4: Asset ownership		E	E	E	E	E	E
Earns a cash income, % (n)^b^	28.1 (541)	E	E	E	E	E	E
Owns house alone or jointly, % (n)	39.5 (761)	-0.091	-0.015	0.022	-0.003	**0.827**	0.013
Owns land alone or jointly, % (n)	32.9 (633)	0.033	0.030	0.000	0.006	**0.998**	-0.004
Dimension 5: Role in HH decision-making							
Own healthcare, % (n)	67.6 (1301)	0.099	-0.031	**0.831**	-0.022	0.004	0.008
HH purchases, % (n)	52.3 (1007)	-0.034	0.007	**0.929**	0.013	0.043	-0.024
Visits to family and friends, % (n)	62.1 (1195)	-0.166	0.075	**0.795**	0.060	-0.086	0.042
Use of husband income, % (n)	57.45 (1103)	0.097	-0.046	**0.742**	-0.039	0.081	-0.034
Dimension 6: Rejects IPV							
Goes out without permission, % (n)	55.0 (1059)	-0.035	0.051	0.043	**0.881**	-0.009	-0.029
Burns food, % (n)	72.9 (1404)	0.233	0.030	-0.037	**0.743**	-0.026	0.027
Neglects child, % (n)	48.4 (933)	-0.031	-0.066	-0.006	**0.938**	0.045	-0.046
Refuses sex, % (n)	64.5 (1243)	0.141	-0.016	-0.005	**0.820**	0.010	0.030
Argues with husband, % (n)	53.2 (1025)	-0.001	0.004	0.026	**0.885**	-0.022	0.020

*E* Eliminated. Indicator was eliminated from the final model because its did not meet the pre-established cut-off, *HH* Household, *IPV* Intimate Partner Violence, *SD* Standard Deviation

^a^ A factor loading is the percentage of an indicator’s variability explained by a given factor (empowerment dimension)

^b^ Proportion of respondents who earned any cash income

### Model results

The final model included six empowerment dimensions measured by 21 observed indicators (Table 2) with good model fit (RMSEA = 0.023, CFI = 0.987, TLI = 0.972, SRMR = 0.024). The original six dimensions were retained in the final model; however, three indicators were eliminated because they did not significantly contribute to any dimension: Earns a Cash Income, Age Difference with Partner, Education Difference with Partner.

**Fig. 2:**
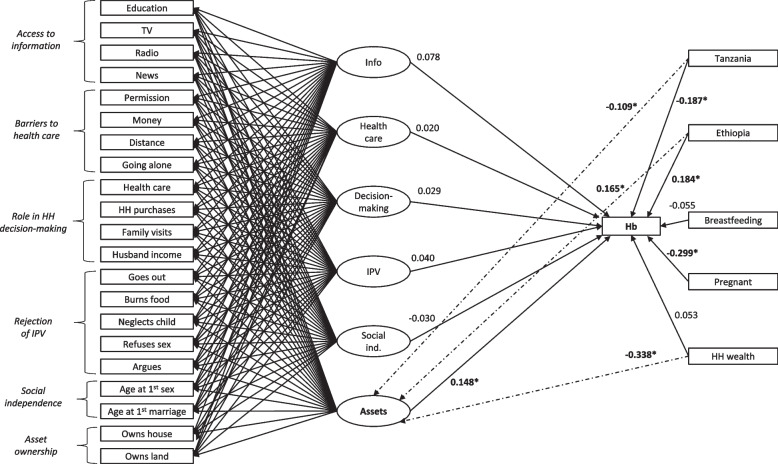
Final exploratory structural equation model of the relationship between married adolescent girls’ haemoglobin levels and empowerment dimensions. Notes: Rectangles indicate observed (directly measured) variables. Ovals represent latent constructs or factors (empowerment dimensions). Unidirectional arrows represent direct effect of exposure on outcome. Values are standardized beta coefficients for respective paths. Bolded values are significant at the 0.05 level. Only statistically significant paths and values are presented. Paths indicated by a dotted line were added to the model following a review of modification indices. Similar paths from household wealth and countries of residence to the remaining empowerment dimensions were statistically significant but are not shown to simplify figure interpretation. Not shown: Error terms for estimated variables, covariance of factors

**Fig. 3:**
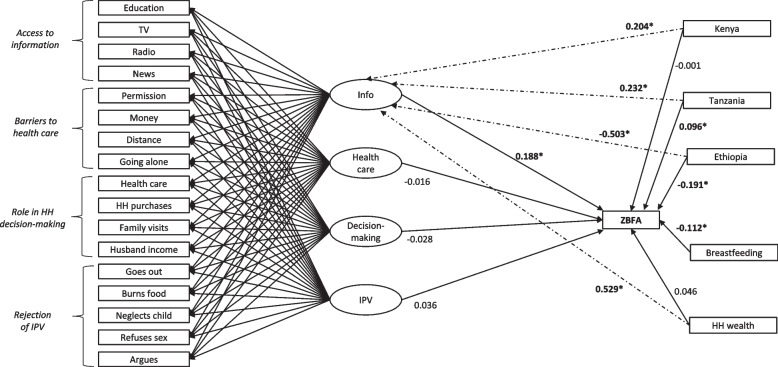
Final exploratory structural equation model of the relationship between married adolescent girls’ BMI-for-age and empowerment dimensions

The final ESEM models for BMI-for-age and haemoglobin are presented in Figs. 2 and 3. The final models included the addition of paths representing the direct influence of household wealth and country of residence on each empowerment dimension based on a review of modification indices and a consideration of their theoretical justification. Model fit results are presented in Additional File 1. Standardized path coefficients for continuous outcomes and adjusted odds ratios (aOR) for binary outcomes with 95% confidence intervals are reported in this section. Unstandardized results are reported in Additional File 1.

#### Haemoglobin

After controlling for household wealth, country of residence, pregnancy, and breastfeeding, one empowerment dimension, asset ownership, had a direct positive association with haemoglobin levels among respondents (0.148, (0.061, 0.235)) (Table 3 and Fig. 2). Expressed in original units, each one unit increase in asset ownership was associated with a 0.262 (0.108, 0.415) increase in haemoglobin (Additional File 1). No other empowerment dimensions were significantly associated with haemoglobin.

**Table 3:** Standardized path coefficients and adjusted odds ratios with 95% confidence intervals for nutrition outcomes by empowerment dimension

Outcome	Access to info	Access to HC	HH decision-making	Rejection of IPV	Social independence	Asset ownership
Hb	0.078 (-0.031, 0.187)	0.020 (-0.060, 0.101)	0.029 (-0.053, 0.110)	0.040 (-0.041, 0.120)	-0.030 (-0.098, 0.037)	**0.148 (0.061, 0.235)**
Anemia^a^	1.005 (0.866, 1.168)	1.015 (0.918, 1.123)	0.966 (0.862, 1.083)	0.989 (0.888, 1.101)	1.046 (0.958, 1.143)	0.928 (0.816, 1.053)
Mod.-sev. anemia^a^	0.872 (0.730, 1.042)	0.974 (0.863, 1.100)	0.954 (0.843, 1.081)	0.905 (0.796, 1.028)	1.046 (0.951, 1.150)	**0.817 (0.716, 0.933)**
ZBFA	**0.188 (0.075, 0.301)**	-0.016 (-0.104, 0.072)	-0.028 (-0.112, 0.055)	0.036 (-0.044, 0.116)	NA	NA
Overweight or obese^a^	1.203 (0.976, 1.481)	0.989 (0.837, 1.169)	0.913 (0.775, 1.076)	**1.168 (1.026, 1.330)**	NA	NA
Thinness^a^	**0.**558^b^	1.088^b^	1.079^b^	1.184^b^		

*95%CI* 95% confidence interval, *Hb* Haemoglobin (g/dL), *HC* Healthcare, *HH* Household, *IPV* Intimate Partner Violence*NA* Not Applicable, *ZBFA* BMI-for-age z-score

Bolded = *p* < 0.05

^a^ Adjusted odds ratio

^b^ Confidence intervals too wide to report here

#### Anemia

Asset ownership was significantly associated with reduced odds of being moderately to severely anemic (aOR = 0.817 (0.716, 0.933)) but the relationship was non-significant (*p* > 0.05) between all empowerment dimensions and anemia when mild cases were included in the model. No other empowerment dimensions were significantly associated with anemia.

#### BMI-for-age

The final BMI-for-age model included only four empowerment dimensions: access to information, access to healthcare, role in household decision-making, and rejection of IPV. The remaining empowerment domains – social independence and asset ownership – were removed from the model because they produced negative residual variances. Consequently, access to information was the only empowerment domain directly and positively associated with BMI-for-age (0.188, 95% CI = 0.075, 0.301)) among non-pregnant respondents after controlling for household wealth, breastfeeding status, and country of residence (Table 3 and Fig. 3). Expressed in original units, a one unit increase in access to information was associated with a 0.113 increase in respondents’ BMI-for-age z-score. The remaining empowerment domains were non-significant.

#### Overweight and obesity

Rejecting justifications for IPV was directly and positive associated with the odds of being overweight or obese (aOR = 1.168, (1.026, 1.330)) among non-pregnant respondents, but the sample size was small (only 103 respondents (7%) were overweight or obese). No other empowerment dimensions were significantly associated with being overweight or obese.

#### Thinness

The relationships between thinness and all empowerment dimensions were non-significant after controlling for country of residence, household wealth, and breastfeeding status in non-pregnant respondents. These results should be interpreted with caution due to the small number of respondents who were thin (*n* = 52 (3.5%)).

## Discussion

The relationship between adolescent girls’ empowerment and their nutritional status is understudied. This study found a statistically significant association between specific dimensions of married adolescent girls’ empowerment and their nutritional status in East Africa using a theoretically informed model of the empowerment-nutrition relationship. Owning assets (a house or land) either alone or jointly was significantly and positively associated with haemoglobin and lower odds of being moderately to severely anemic. Access to information (measured by girls’ level of completed education and their mass media use) was significantly and positively associated with BMI-for-age. Rejection of justifications for IPV was associated with the odds of being overweight or obese. No empowerment dimensions were significantly associated with being thin, however the sample size was small. The empowerment dimensions of access to healthcare, role in household decision-making, and social independence were not significantly associated with any assessed nutrition outcomes.

The positive relationships between asset ownership and access to information with haemoglobin and BMI-for-age, respectively, suggest that access to economic and educational resources play an important role in the empowerment-nutrition relationship. In settings where female empowerment is far from complete, such as East Africa, measures of resource access and control may be just as informative as direct measures of agency and may be critical to understanding the process of empowerment [23, 48]. Married adolescent girls who (co-)own a house or land, particularly in places where agriculture is the main source of economy for women, may have greater access to resources in general that can translate into access to a good diet and healthcare. Higher education and mass media use may increase adolescent girls’ exposure to nutrition- and diet-related information and indirectly affect their nutritional status by reducing adolescent fertility and delaying marriage and childbearing. On the other hand, peer influence, the changing food environment, and mass/social media can promote unhealthy nutrition-related behaviours, such as increased consumption of ultra-processed foods, as well as unhealthy conceptualizations of body image [13, 49]. Rejection of justifications of IPV, a measure of girls’ intrinsic agency, was associated with increased odds of being overweight or obese in our study population. A possible explanation is that girls with higher levels of intrinsic agency have greater freedom to exercise choice in their diet and may therefore choose to eat foods that are popular or convenient but may be lacking in nutritional value.

We did not find any significant associations between nutrition outcomes and adolescent girls’ social independence. Age at first marriage and first sex (the indicators used to measure social independence) have been previously shown to be associated with nutrition; women who delay marriage and childbearing generally have better maternal and child health and nutrition outcomes. It may be that the absence of important mediating variables along the causal chain from social independence to nutritional status, such as level of education, are needed to help explain the relationship.

Girls’ ability to overcome barriers to healthcare access was not significantly associated with their BMI-for-age or haemoglobin. We hypothesized that given our study population was generally healthy (mean Hb = 12.0 g/dL, mean BMI = 20.8 kg/m^2^), they may not regularly seek healthcare, making it difficult to detecting a statistical relationship with nutritional status.

Recent studies have identified three- and four-factor models for women’s empowerment in sub-Saharan Africa that resemble our dimensions for social independence, role in household decision-making, and rejection of IPV [32, 50–52]. Jones and colleagues used structural equation modelling to assess the relationship between married women’s (15–49) empowerment and nutritional status in five East African countries [21]. Their model included the three domains listed above and found significant positive associations between all three domains and women’s BMI, and between role in household decision-making and haemoglobin. We were unable to identify a statistically significant association between household decision-making and adolescent girls’ nutrition, but this measure may be less meaningful for married adolescent girls as they are less likely to hold sway in the household compared to adult women, their husbands, and in-laws.

There is limited understanding of how variations in age and context affect girls’ empowerment [12]. While our data included older adolescents only, we were able to explore the influence of selected contextual factors on empowerment and nutrition. Our initial model did not include pathways from covariates representing household wealth and country of residence to empowerment dimensions, but the models’ modifications indices recommended the addition of these paths to reflect the significant relationship between these contextual measures and empowerment. Context-specific structural characteristics, such as socio-economic status and gendered norms, beliefs, and practices, can constrain girls’ choices and influence their behaviour, values, and preferences. Geographic measures, such as country of residence, can act as proxy indicators for engrained rules, norms, and practices that shape girls’ agency [23]. Others have similarly found that levels of empowerment vary by country and household wealth [21, 50, 53]. Existing models of adolescent empowerment recognize that supportive laws, policies, structures, and adults/decision-makers are a crucial influence on pathways from adolescent empowerment to improved health and well-being [11, 12, 54]. These relationships undoubtedly vary by household, community, region, and state, and must be examined when seeking to design and measure meaningful empowerment-related change. In East Africa, continued high rates of HIV infection among adolescents, especially girls, is an important contextual factor given the intersection of the HIV/AIDS epidemic and adolescent health seeking behaviour in this region [55].

Our greatest limitation was data availability. There are few choices for researchers seeking datasets that include both gender-related and health-related data. The DHS is one of the few options available, but it is not designed for analyses of adolescent populations and does not collect data for younger adolescents (aged 10–14 years), nor does it collect the women’s status module for unmarried women and adolescents. We were unable to test single country models because of small country sample sizes for our target population, and our results suggest that this would be meaningful given country of residence is directly related to empowerment. The DHS also lacks measures for other potentially important empowerment dimensions and confounders of the nutrition-empowerment relationship. We included as many relevant and available empowerment indicators and covariates as possible and maximized our sample by pooling country datasets to form a regional sample to overcome these limitations. However, we still had two dimensions measured by only two indicators, which led to the elimination of both dimensions in the BMI-for-age model because of model instability. One benefit of using DHS data is that this model can be used to test the association of dimensions of empowerment with other health outcomes and across contexts due to its standardized format that allows cross-country and -time comparisons.

Our findings suggest that investment in girls’ access to information through education and mass/social media remains an important strategy for their empowerment and nutrition. Others have similarly argued that investing in secondary education, particularly for girls, provides extensive opportunities for health and well-being. [56] This will require investments in interventions that address some of the greatest gender-related barriers to girls’ education, including restrictive gender norms that devalue girls’ education, cultural practices of child/early/forced marriage and early pregnancy, and inadequate water and sanitation facilities in schools. Schools can also serve as important platforms for health promotion and services, such as nutrition education and iron and folic acid supplementation [11].

Recent evidence points to the value of social media for addressing overnutrition. Increasing rates of overweight and obesity in the region signal the need to invest in these areas as well. However, low access to digital technology in the region could be an impediment [57]. Caution should also be exercised given the potentially negative influence of social media on body image and the ability of industry to directly market unhealthy food and beverage choices to adolescents through various media platforms [7, 49].

The economic empowerment of adolescent girls is receiving increasing attention, whether it is through examining gender disparities in economic opportunities between adolescent boys and girls [58], or the incorporation of economic empowerment strategies in adolescent nutrition interventions, such as income generating activities, savings and loan program, and life skills training [59, 60]. Our findings suggest that adolescent girls’ economic empowerment may influence their haemoglobin levels, but we could not test this relationship for BMI-for-age due to modelling limitations. Others have found equivocal results on the economic empowerment and nutrition link for adolescent girls as well [59]. More investigation is warranted.

There is an urgent need for better data on adolescent empowerment and health to facilitate research on the gendered pathways to better health for adolescents. This includes the need for datasets to be sufficiently powered to analyse other socially stratifying factors such as ethnicity and disability, and more balanced data that captures gender-related measures for boys and girls [58, 59, 61, 62]. Much work remains to further our understanding of the mechanisms of action that link adolescent girls’ empowerment and nutrition. Studies that assess causality and further our options for validated measures across contexts are needed. This latter point cannot replace the importance of understanding the role of context in the empowerment process, such as the reasons behind varying levels of empowerment by country. The use of statistical models should be complemented by robust qualitative research to further results interpretation.

## Supplementary Information

**Additional file 1:** **Table A1.** Model indicator descriptions and coding. **Table A2.** Unstandardized path coefficients with 95% confidence intervals for nutrition outcomes by empowerment dimension. **Table A3.** Standardized path coefficients with 95% confidence intervals for nutrition outcomes by empowerment dimension. **Table A4.** Model fit results. **Table A5.** Standardized path coefficients and 95% confidence intervals for nutrition outcomes by empowerment domain for models including additional covariates. **Table A6.** Model fit results for models with additional covariates. **Table A7.** Standardized path coefficients and 95% confidence intervals for the direct association of model covariates and empowerment dimensions.

## Data Availability

The datasets analysed during the current study are available from the Demographic and Health Survey Program, https://dhsprogram.com/Data/.

## Abbreviations

BMI: Body mass index
CFA: Confirmatory factor analysis
CFI: Comparative fit index
CI: Confidence interval
DHS: Demographic and Health Survey
EFA: Exploratory factor analysis
ESEM: Exploratory structural equation model
Hb: Haemoglobin
HIV: Human immunodeficiency virus
IPV: Intimate partner violence
IRB: Institutional Review Board
RMSEA: Root mean square error of approximation
SD: Standard deviation
SEM: Structural equation model
SRMR: Standardized root mean square residual
TLI: Tucker-Lewis index
UGM: Unweighted grand mean
WHO: World Health Organization
WLSMV: Variance-adjusted weighted least squares
ZBFA: BMI-for-age z-score
