# Supplementary material for: Associations between dimensions of empowerment and nutritional status among married adolescent girls in East Africa: a structural equation modelling study
Source: BMC Public Health. 2023 Feb 2;23:225. doi: 10.1186/s12889-022-14949-1 (PMC9893589; doi:10.1186/s12889-022-14949-1)
Supplement: Supplementary file 1 — Additional file 1: Table A1. Model indicator descriptions and coding. Table A2. Unstandardized path coefficients with 95% confidence intervals for nutrition outcomes by empowerment dimension. Table A3. Standardized path coefficients with 95% confidence intervals for nutrition outcomes by empowerment dimension. Table A4. Model fit results. Table A5. Standardized path coefficients and 95% confidence intervals for nutrition outcomes by empowerment domain for models including additional covariates. Table A6. Model fit results for models with additional covariates. Table A7. Standardized path coefficients and 95% confidence intervals for the direct association of model covariates and empowerment dimensions. [file 12889_2022_14949_MOESM1_ESM.docx]

Additional File 1

Table of Contents

[List of selected empowerment studies using DHS data 2](#_Toc121125948)

[Table A1. Model indicator descriptions and coding. 4](#_Toc121125949)

[Table A2. Unstandardized path coefficients with 95% confidence intervals for nutrition outcomes by empowerment dimension. 10](#_Toc121125950)

[Table A3. Standardized path coefficients with 95% confidence intervals for nutrition outcomes by empowerment dimension. 11](#_Toc121125951)

[Table A4. Model fit results. 12](#_Toc121125952)

[Table A5. Standardized path coefficients and 95% confidence intervals for nutrition outcomes by empowerment domain for models including additional covariates 13](#_Toc121125953)

[Table A6. Model fit results for models with additional covariates. 13](#_Toc121125954)

[Table A7. Standardized path coefficients and 95% confidence intervals for the direct association of model covariates and empowerment dimensions. 14](#_Toc121125955)

## List of selected empowerment studies using DHS data

1. Asaolu, I. O., Alaofè, H., Gunn, J. K. L., Adu, A. K., Monroy, A. J., Ehiri, J. E., Hayden, M. H., & Ernst, K. C. (2018). Measuring Women’s Empowerment in Sub-Saharan Africa: Exploratory and Confirmatory Factor Analyses of the Demographic and Health Surveys. Frontiers in Psychology, 9. https://doi.org/10.3389/fpsyg.2018.00994
2. Asaolu, I. O., Okafor, C. T., Ehiri, J. C., Dreifuss, H. M., & Ehiri, J. E. (2017). Association between Measures of Women’s Empowerment and Use of Modern Contraceptives: An Analysis of Nigeria’s Demographic and Health Surveys. Frontiers in Public Health, 4. https://doi.org/10.3389/fpubh.2016.00293
3. Ballon, P. (2018). A Structural Equation Model of Female Empowerment. The Journal of Development Studies, 54(8), 1303–1320. https://doi.org/10.1080/00220388.2017.1414189
4. Doku, D. T., Bhutta, Z. A., & Neupane, S. (2020). Associations of women’s empowerment with neonatal, infant and under-5 mortality in low- and /middle-income countries: meta-analysis of individual participant data from 59 countries. BMJ Global Health, 5(1), e001558. https://doi.org/10.1136/bmjgh-2019-001558
5. Ewerling, F., Lynch, J. W., Victora, C. G., van Eerdewijk, A., Tyszler, M., & Barros, A. J. D. (2017). The SWPER index for women’s empowerment in Africa: development and validation of an index based on survey data. The Lancet Global Health, 5(9), e916–e923. https://doi.org/10.1016/S2214-109X(17)30292-9
6. Ewerling, F., Raj, A., Victora, C. G., Hellwig, F., Coll, C. V. N., & Barros, A. J. D. (2020). SWPER Global: A survey-based women’s empowerment index expanded from Africa to all low- and middle-income countries. JOURNAL OF GLOBAL HEALTH, 10(2). https://doi.org/10.7189/jogh.10.020434
7. Jennings, L., Na, M., Cherewick, M., Hindin, M., Mullany, B., & Ahmed, S. (2014). Women’s empowerment and male involvement in antenatal care: analyses of Demographic and Health Surveys (DHS) in selected African countries. BMC Pregnancy and Childbirth, 14(1), 297. https://doi.org/10.1186/1471-2393-14-297
8. Jones, R. E., Haardörfer, R., Ramakrishnan, U., Yount, K. M., Miedema, S. S., Roach, T. D., & Girard, A. W. (2020). Intrinsic and instrumental agency associated with nutritional status of East African women. Social Science & Medicine, 247, 112803. https://doi.org/10.1016/j.socscimed.2020.112803
9. Jones, R., Haardörfer, R., Ramakrishnan, U., Yount, K. M., Miedema, S., & Girard, A. W. (2019). Women’s empowerment and child nutrition: The role of intrinsic agency. SSM - Population Health, 9, 100475. https://doi.org/10.1016/j.ssmph.2019.100475
10. Kabir, A., Rashid, M. M., Hossain, K., Khan, A., Sikder, S. S., & Gidding, H. F. (2020). Women’s empowerment is associated with maternal nutrition and low birth weight: evidence from Bangladesh Demographic and Health Survey. BMC Women’s Health, 20(93).
11. Kishor, S., & Subaiya, L. (2008). Understanding Women’s Empowerment: A Comparative Analysis of Demographic and Health Surveys (DHS) Data. In DHS Comparative Reports No. 20.
12. Miedema, S. S., Haardörfer, R., Girard, A. W., & Yount, K. M. (2018). Women’s empowerment in East Africa: Development of a cross-country comparable measure. World Development, 110, 453–464. https://doi.org/10.1016/j.worlddev.2018.05.031
13. Na, M., Jennings, L., Talegawkar, S. A., & Ahmed, S. (2015). Association between women’s empowerment and infant and child feeding practices in sub-Saharan Africa: an analysis of Demographic and Health Surveys. Public Health Nutrition, 18(17), 3155–3165. https://doi.org/10.1017/S1368980015002621
14. Pratley, P., & Sandberg, J. F. (2018). Refining the Conceptualization and Measurement of Women’s Empowerment in Sub-Saharan Africa Using Data from the 2013 Nigerian Demographic and Health Survey. Social Indicators Research, 140(2), 777–793. https://doi.org/10.1007/s11205-017-1811-1
15. Sebayang, S. K., Efendi, F., & Astutik, E. (2017). Women’s empowerment and the use of antenatal care services in Southeast Asian countries. In DHS Working Papers No. 129. http://dhsprogram.com/pubs/pdf/WP129/WP129.pdf
16. Shimamoto, K., & Gipson, J. D. (2017). Examining the mechanisms by which women’s status and empowerment affect skilled birth attendant use in Senegal: a structural equation modeling approach. BMC Pregnancy and Childbirth, 17(S2), 341. <https://doi.org/10.1186/s12884-017-1499-x>
17. Shimamoto, K., & Gipson, J. D. (2019). Investigating pathways linking women’s status and empowerment to skilled attendance at birth in Tanzania: A structural equation modeling approach. PLOS ONE, 14(2), e0212038. <https://doi.org/10.1371/journal.pone.0212038>
18. Yaya, S., Odusina, E. K., Uthman, O. A., & Bishwajit, G. (2020). What does women’s empowerment have to do with malnutrition in Sub-Saharan Africa? Evidence from demographic and health surveys from 30 countries. Global Health Research and Policy, 5(1), 1. <https://doi.org/10.1186/s41256-019-0129-8>

## Table A1. Model indicator descriptions and coding.

| **DHS variable name** | **DHS variable description** | **Recode name** | **Recode description** |
| --- | --- | --- | --- |
| v133 | Education in single years  Constructed from the education level (v106) and the grade at that level (v107) | educ | Recoded to replace zero values with missing "." |
| V157 | Frequency of reading newspaper or magazine  0 = "Not at all"  1 = "Less than once a week"  2 = "At least once a week"  3 = "Almost every day" | News | 1 = reads less than once per week or more 0 = not at all |
| v158 | Frequency of listening to radio  0 = "Not at all" 1 = "Less than once a week" 2 = "At least once a week" 3 = "Almost every day" | Radio | 1 = listens less than once per week or more 0 = not at all |
| v159 | Frequency of watching TV  0 = "Not at all"  1 = "Less than once a week"  2 = "At least once a week"  3 = "Almost every day" | TV | 1 = watches less than once per week or more 0 = not at all |
| v190 | Wealth index  1 = "Poorest" 2 = "Poorer" 3 = "Middle" 4 = "Richer" 5 = "Richest" | -- | -- |
| v191 | Wealth index factor score (5 decimals) | wealth | Recoded to add 5 decimal spaces |
| v213 | Currently pregnant  0 = "No or unsure"  1 = "Yes" | -- | -- |
| v404 | Currently breastfeeding  0 = No 1 = Yes | -- | -- |
| v437 | Respondent's weight (kg) (1 decimal) | wgt | Recoded to add 1 decimal place |
| v438 | Respondent's height (cm) (1 decimal) | hgt | Recoded to add 1 decimal place |
| v445 |  | CBMI | Calculated using AnthroPlus |
|  |  | ZBFA | Calculated using AnthroPlus |
|  |  | thin | 1 = "Thin" (ZBFA <= -2) 0 = "Not thin" (ZBFA > -2) |
|  |  | overwgt | 1 = "Overweight or obese" (ZBFA > 1) 0 = "Not overweight or obese" (ZBFA <= 1) |
| v456 | Hb level adjusted for altitude and smoking (g/dL) ( 1decimal) | hb | Recoded to add 1 decimal place |
| v457 | Anemia status  1 = "Severe" 2 = "Moderate" 3 = "Mild" 4 = "Not anemic"  Any anemia:  · Number of non-pregnant women whose hemoglobin count is less than 12.0 grams per deciliter (g/dl) (v456 < 120)  · plus number of pregnant women whose count is less than 11.0 g/dl (v456 < 110)  2) Mild anemia:  · Number of non-pregnant women whose hemoglobin count is between 11.0 and 11.9 g/dl (v456 in 110:119)  · plus number of pregnant women whose hemoglobin count is between 10.0 and 10.9 g/dl (v456 in 100:109)  3) Moderate anemia:  · Number of non-pregnant women whose hemoglobin count is between 8.0 and 10.9 g/dl (v456 in 80:109)  · Number of pregnant women whose hemoglobin count is between 7.0 and 9.9 g/dl (v456 in 70:99)  4) Severe anemia:  · Number of non-pregnant women whose hemoglobin count is less than 8.0 g/dl (v456<80)  · Number of pregnant women whose hemoglobin count is less than 7.0 g/dl (v456<70) | Anemia | 1 = (v456 < 120) or (v456 < 110 for pregnant respondents) 0 = Not anemic |
|  |  | Mod to sev anemia | 1 = (v456 < 110) or (v456 < 100 for pregnant respondents) 0 = Not moderately to severely anemic |
| v467b | Permission is a barrier to health care for self  0 = "No problem"  1 = "Big problem"  2 = "Not a big problem" | permis | 1 = No problem or not a big problem 0 = big problem |
| v467c | Money is a barrier to health care for self  0 = "No problem"  1 = "Big problem"  2 = "Not a big problem" | money | 1 = No problem or not a big problem 0 = big problem |
| v467d | Distance is a barrier to health care for self  0 = "No problem"  1 = "Big problem"  2 = "Not a big problem" | distance | 1 = No problem or not a big problem 0 = big problem |
| v467f | Going alone is a barrier to health care for self  0 = "No problem"  1 = "Big problem"  2 = "Not a big problem" | alone | 1 = No problem or not a big problem 0 = big problem |
| v511 | Age at first union | -- | -- |
| v531 | Age at first sex (imputed) | -- | -- |
| v715 | Partner education level | -- | -- |
| v730 | Partner age | -- | -- |
| v741 | Type of earnings  0 = "Not paid"  1 = "Cash only"  2 = "Cash and in-kind"  3 = "In-kind only" | earnings | 2 = Cash and in-kind or cash only 1 = in-kind only 0 = not paid |
| v743a | Person who decides on health care  1 = "Respondent alone"  2 = "Respondent and husband/partner"  3 = "Respondent and other person"  4 = "Husband/partner alone"  5 = "Someone else"  6 = "Other" | health | 1 = Decides alone or jointly (with husband or other) 0 = no role in decision-making |
| v743b | Person who decided on large HH purchases  1 = "Respondent alone"  2 = "Respondent and husband/partner"  3 = "Respondent and other person"  4 = "Husband/partner alone"  5 = "Someone else"  6 = "Other" | purchase | 1 = Decides alone or jointly (with husband or other) 0 = no role in decision-making |
| v743d | Person who decides on visits to friends and family  1 = "Respondent alone"  2 = "Respondent and husband/partner"  3 = "Respondent and other person"  4 = "Husband/partner alone"  5 = "Someone else"  6 = "Other" | visit | 1 = Decides alone or jointly (with husband or other) 0 = no role in decision-making |
| v743f | Person who decided on husband's income  1 = "Respondent alone"  2 = "Respondent and husband/partner"  3 = "Respondent and other person"  4 = "Husband/partner alone"  5 = "Someone else"  6 = "Other"  7 = "Husband/partner has no earnings" | husband | 1 = Decides alone or jointly (with husband or other) 0 = no role in decision-making |
| v744a | Going out without tell husband is justification for violence  0 = "No"  1 = "Yes"  8 = "Don't know" | out | 1 = No 0 = Yes or don't know |
| v744b | Neglecting children is justification for violence  0 = "No"  1 = "Yes"  8 = "Don't know" | neglect | 1 = No 0 = Yes or don't know |
| v744c | Arguing with husband is justification for violence  0 = "No"  1 = "Yes"  8 = "Don't know" | argue | 1 = No 0 = Yes or don't know |
| v744d | Refusing sex is justification for violence  0 = "No"  1 = "Yes"  8 = "Don't know" | refuse | 1 = No 0 = Yes or don't know |
| v744e | Burning food is justification for violence  0 = "No"  1 = "Yes"  8 = "Don't know" | burns | 1 = No 0 = Yes or don't know |
| v745a | Owns a house | house | 1 = Owns house alone or jointly 0 = Does not own |
| v745b | Owns land | land | 1 = Owns land alone or jointly 0 = Does not own |
| -- | -- | country | 11 = "Ethiopia" 22 = "Tanzania" 33 = "Kenya" 44 = "Uganda" 55 = "Rwanda" |

## Table A2. Unstandardized path coefficients with 95% confidence intervals for nutrition outcomes by empowerment dimension.

| Outcome | N | Access to info | Access to HC | HH decision-making | Rejection of IPV | Social Independence | Asset ownership |
| --- | --- | --- | --- | --- | --- | --- | --- |
| Haemoglobin (g/dL) | 1575 | 0.111  (-0.042, 0.264) | 0.036  (-0.106, 0.178) | 0.050  (-0.092, 0.192) | 0.074  (-0.077, 0.225) | -0.056  (-0.182, 0.069) | **0.262**  **(0.108, 0.415)** |
| Anemia^a^ | 1575 | 0.004  (-0.116, 0.124) | 0.015  (-0.086, 0.116) | -0.034  (-0.034, -0.147) | -0.012  (-0.125, 0.101) | 0.048  (-0.045, 0.141) | -0.075  (-0.202, 0.051) |
| Mod.-sev. anemia^b^ | 1575 | -0.108  (-0.248, 0.032) | -0.026  (-0.145, 0.093) | -0.046  (-0.166, 0.075) | -0.104  (-0.237, 0.029) | 0.046  (-0.052, 0.145) | **-0.198**  **(-0.327, -0.069)** |
| BMI-for-age  (z-score) | 1511 | **0.113**  **(0.045, 0.181)** | -0.012  (-0.080, 0.055) | -0.023  (-0.092, 0.045) | 0.031  (-0.037, 0.099) | NA | NA |
| Overweight^c^ | 1511 | 0.134  (-0.018, 0.286) | -0.010  (-0.165, 0.144) | -0.090  (-0.253, 0.072) | **0.161**  **(0.028, 0.293)** | NA | NA |
| Thinness^d^ | 1511 | -0.412  (-1.181, 0.0357) | 0.075  (-0.239, 0.389) | 0.073  (-0.339, 0.486) | 0.170  (-0.555, 0.894) | NA | NA |

Bolded = p < 0.05.

BMI = Body mass index.

CI = Confidence Interval.

HC = Health Care.

HH = Household.

IPV = Intimate Partner Violence.

NA = Not Available.

^a^ Hb < 12.0 g/dl for non-pregnant, Hb < 11.0 for pregnant.

^b^ Hb < 11.0 g/dl for non-pregnant, H1 < 10.0 for pregnant.

^c^ ZBFA > 1SD.

^d^ ZBFA < -2SD.

## Table A3. Standardized path coefficients with 95% confidence intervals for nutrition outcomes by empowerment dimension.

| Outcome | N | Access to info | Access to HC | HH decision-making | Rejection of IPV | Social Independence | Asset ownership |
| --- | --- | --- | --- | --- | --- | --- | --- |
| Haemoglobin (g/dL) | 1575 | 0.078  (-0.031, 0.187) | 0.020  (-0.060, 0.101) | 0.029  (-0.053, 0.110) | 0.040  (-0.041, 0.120) | -0.030  (-0.098, 0.037) | **0.148**  **(0.061, 0.235)** |
| Anemia^a^ | 1575 | 0.005  (-0.144, 0.155) | 0.015  (-0.086, 0.116) | -0.035  (-0.149, 0.080) | -0.011  (-0.119, 0.096) | 0.045  (-0.043, 0.134) | -0.075  (-0.203, 0.052) |
| Mod.-sev. anemia^b^ | 1575 | -0.137  (-0.315, 0.041) | -0.026  (-0.147, 0.095) | -0.047  (-0.171, 0.078) | -0.100  (-0.228, 0.028) | 0.045  (-0.050, 0.140) | **-0.202**  **(-0.334, -0.069)** |
| BMI-for-age  (z-score) | 1511 | **0.188**  **(0.075, 0.301)** | -0.016  (-0.104, 0.072) | -0.028  (-0.112, 0.055) | 0.036  (-0.044, 0.116) | NA | NA |
| Overweight^c^ | 1511 | 0.185  (-0.024, 0.393) | -0.011  (-0.178, 0.156) | -0.091  (-0.255, 0.073) | **0.155**  **(0.026, 0.285)** | NA | NA |
| Thinness^d^ | 1511 | -0.583  (-199.532, 198.366) | 0.084  (-28.414, 28.581) | 0.076  (-25.414, 26.070) | 0.169  (-57.386, 57.723) | NA | NA |

Bolded = p < 0.05.

BMI = Body mass index.

CI = Confidence Interval.

HC = Health Care.

HH = Household.

IPV = Intimate Partner Violence.

NA = Not Available.

^a^ Hb < 12.0 g/dl for non-pregnant, Hb < 11.0 for pregnant.

^b^ Hb < 11.0 g/dl for non-pregnant, H1 < 10.0 for pregnant.

^c^ ZBFA > 1SD.

^d^ ZBFA < -2SD.

## Table A4. Model fit results.

| Model | Chi-square value (df) p-value | RMSEA | CFI | TLI | SRMR |
| --- | --- | --- | --- | --- | --- |
|  |  |  |  |  |  |
| BMI-for-age | 436.624 (156) 0.0000 | 0.035 | 0.954 | 0.929 | 0.049 |
| Haemoglobin | 437.478 (201) 0.0000 | 0.027 | 0.966 | 0.942 | 0.042 |
| Anemia | 435.596 (201) 0.0000 | 0.027 | 0.966 | 0.942 | 0.043 |
| Mod to sev anemia | 439.189 (201) 0.0000 | 0.027 | 0.965 | 0.941 | 0.043 |
| Overweight | 430.124 (156) 0.0000 | 0.034 | 0.955 | 0.930 | 0.049 |
| Thin | 353.129 (156) 0.0000 | 0.029 | 0.965 | 0.945 | 0.081 |

BMI = Body mass index.

CFI = Confirmatory Factor Index.

df = Degrees of freedom.

RMSEA = Root Mean Square Error Approximation.

SRMR = Standardized Root Mean Square Residual.

TLI = Tucker Lewis Index.

Table A5. Standardized path coefficients and 95% confidence intervals for nutrition outcomes by empowerment domain for models including additional covariates.^[[1]](#footnote-1)^

| Outcome | Access to info | Access to HC | HH decision-making | Rejection of IPV | Social independence | Asset ownership |
| --- | --- | --- | --- | --- | --- | --- |
| Hb | 0.074 | 0.019 | 0.033 | 0.054 | -0.053 | **0.138** |
| 95%CI | (-0.038, 0.187) | (-0.062, 0.101) | (-0.053, 0.119) | (-0.025, 0.133) | (-0.129, 0.023) | **(0.049, 0.227)** |
| ZBFA | **0.212** | -0.027 | -0.027 | 0.046 | -0.074 | NA |
| 95%CI | **(0.099, 0.324)** | (-0.118, 0.063) | (-0.108, 0.053) | (-0.035, 0.127) | (-0.154, 0.006) |  |

Bolded = p < 0.05.

CI = Confidence interval.

Hb = Haemoglobin (g/dL).

HC = Health care.

HH = Household.

IPV = Intimate Partner Violence.

NA = Not Available.

ZBFA = BMI-for-age z-score.

## Table A6. Model fit results for models with additional covariates.

| Model | Chi-square value (df) p-value | RMSEA | CFI | TLI | SRMR |
| --- | --- | --- | --- | --- | --- |
|  |  |  |  |  |  |
| BMI-for-age | 963.698 (251) 0.0000 | 0.036 | 0.909 | 0.866 | 0.066 |
| Haemoglobin | 986.075 (264) 0.0000 | 0.036 | 0.919 | 0.874 | 0.074 |

BMI = Body mass index.

CFI = Confirmatory Factor Index.

df = Degrees of freedom.

RMSEA = Root Mean Square Error Approximation.

SRMR = Standardized Root Mean Square Residual.

TLI = Tucker Lewis Index.

## Table A7. Standardized path coefficients and 95% confidence intervals for the direct association of model covariates and empowerment dimensions.

|  | **F1**  **Info** | **F2**  **Health care** | **F3**  **Decisions** | **F4**  **IPV** | **F5**  **Assets** | **F6**  **Soc. Indep.** |
| --- | --- | --- | --- | --- | --- | --- |
| **Lives in Ethiopia** |  |  |  |  |  |  |
| Hb model | -0.462  (-0.531 to -0.393) | -0.237  (-0.318 to -0.155) | 0.372  (0.291 to 0.454) | -0.097  (-0.186 to -0.008) | -0.089  (-0.178 to 0.000) | 0.165  (0.073 to 0.258) |
| ZBFA model | -0.503  (-0.578 to -0.429) | -0.373  (-0.458 to -0.288) | 0.379  (0.288 to 0.470) | -0.126  (-0.224 to -0.028) | NA | NA |
| **Lives in Kenya** |  |  |  |  |  |  |
| Hb model | NA | NA | NA | NA | NA | NA |
| ZBFA model | 0.204  (0.140 to 0.268) | 0.281  (0.216 to 0.346) | -0.044  (-0.121 to 0.032) | 0.131  (0.053 to 0.209) | NA | NA |
| **Lives in Tanzania** |  |  |  |  |  |  |
| Hb model | 0.385  (0.324 to 0.446) | -0.007  (-0.082 to 0.069) | -0.242  (-0.310 to -0.174) | -0.091  (-0.161 to -0.020) | -0.064  (-0.133 to 0.006) | -0.109  (-0.195 to -0.022) |
| ZBFA model | 0.232  (0.167 to 0.297) | -0.107  (-0.175 to -0.038) | -0.218  (-0.289 to -0.147) | -0.164  (-0.236 to -0.092) | NA | NA |
| **Lives in Uganda** |  |  |  |  |  |  |
| Hb model | 0.077 | 0.244 | -0.130 | 0.188 | 0.153 | -0.056 |
| ZBFA model | 0.067 | 0.199 | -0.117 | 0.159 | NA | NA |
| **Household wealth** |  |  |  |  |  |  |
| Hb model | 0.444  (0.377 to 0.511) | 0.292  (0.214 to 0.370) | 0.086  (0.020 to 0.152) | 0.138  (0.074 to 0.201) | -0.338  (-0.416 to -0.259) | 0.165  (0.086 to 0.224) |
| ZBFA model | 0.529  (0.474 to 0.584) | 0.292  (0.232 to 0.353) | 0.049  (-0.016 to 0.114) | 0.157  (0.088 to 0.226) | NA | NA |

Hb = Haemoglobin.

NA = Not available.

ZBFA = BMI-for-age.

The Kenya covariate was omitted from the Hb model because anemia testing was not conducted during the 2014 Kenya DHS.

1. Additional covariates are: (1) total number of children ever born, (2) household’s main source of drinking water, (3) total number of household members, (4) type of place of residence (urban, rural). [↑](#footnote-ref-1)
